# Avian Influenza—Factors Affecting Consumers’ Purchase Intentions toward Poultry Products

**DOI:** 10.3390/ijerph16214139

**Published:** 2019-10-28

**Authors:** Xiaowei Wen, Sangluo Sun, Lin Li, Qinying He, Fu-Sheng Tsai

**Affiliations:** 1 College of Economics and Management, South China Agricultural University, Guangzhou 510642, China; wxwcn@scau.edu.cn (X.W.); sunsangluo@stu.scau.edu.cn (S.S.); heqy83@scau.edu.cn (Q.H.); 2 Youth Volunteers’ Guidance Center of Guangzhou, Guangzhou 510642, China; 3 Department of Business Administration, Cheng Shiu University, Kaohsiung 83347, Taiwan; 4 Center for Environmental Toxin and Emerging-Contaminant Research, Cheng Shiu University, Kaohsiung 83347, Taiwan; 5 Super Micro Mass Research and Technology Center, Cheng Shiu University; Kaohsiung 83347, Taiwan

**Keywords:** consumption, avian influenza, purchase intentions, media, China

## Abstract

Recently, continuing outbreaks of avian influenza in China have not only caused great loss to the agricultural sector but also brought fear and distrust to consumers, seriously undermining consumer confidence in chicken products. We investigated consumers’ purchase intentions during avian influenza outbreaks by examining a regionally representative sample of 330 consumers in Guangzhou. With respect to 7 kinds of attributes, the ordered logit analysis indicated that possible health threat and uncertainty of the origin of poultry products may cause concern among consumers and cause them to avoid purchasing chicken products. Media reports have a great influence on consumers’ intentions to purchase chicken products during avian influenza outbreaks. Overall, this study suggests establishing an effective mechanism of public knowledge (of chicken products’ safety and quality) enhancement, in order to curb misleading media reports during avian influenza outbreaks.

## 1. Introduction

Human food choice is a complex function [1]. Numerous studies have analyzed the influencing factors affecting consumers’ purchase intentions. A multi-attribute model suggesting that a person’s attitude towards an object is determined by the sum of beliefs has been widely used in consumer research [2]. Other experts assumed that people’s life course experience such as ideals, individual characteristics, resources and so on, mainly affected their choices on food [3]. Theories commonly applied to the study of food consumption are attitude-behavior models, such as the Theory of Reasoned Action (TRA) and the Theory of Planned Behavior (TPB), implying that intention is a function of 3 principle determinants—attitude toward the behavior, subjective norm and perceived behavioral control [4,5,6,7]. Previous studies confirmed that these elements are good predictors of consumers’ purchase intentions on foods [8,9,10,11]. Besides, food risk is one of the other determinants affecting people’s purchase intentions [12,13,14]. Public perceptions of both risks and benefits are crucial [15,16]. With a lack of credible and understandable food safety signals or information, consumers face uncertainty and incur specific information search costs [17]. Consumers’ food choices are believed to be greatly influenced by how they judge the available information [18]. Such information is much needed when deciding whether they should purchase foods [19,20]. Previous findings have also focused on general consumer confidence, including optimism and pessimism, the effect on consumer trust and purchase intentions [21,22,23]. The existing literature sheds light on a number of key issues and increases our understanding of purchase intention and its influencing factors toward some important food items, such as meat, raw milk [24,25,26], fish and aquaculture products [27,28,29], genetically modified food or organic food [30,31,32,33] and so on.

In the last few decades, various kinds of animal diseases, such as mad cow disease (Bovine Spongiform Encephalopathy, BSE), avian influenza and so on, have caused multidimensional economic impacts, calling for effective policy response [34,35]. What is more, they have made consumers worried about food safety and quality, which may lead to a reduction of food’s share of the market [36,37,38]. Some experts found that distrust is negative for the consumption of chicken, and reinforced that neither risk-perception reducing information nor risk-perception elevating information are in use to save the decreasing consumption of chicken, possibly because trust in positive information is still not high in China [39]. Adversely, despite BSE incidents being announced in 2003, statistics showed that Canadians trust Canada’s Food Inspection Agency, and they did not think anyone in Canada would become infected. In both 2003 and 2005, Canadian domestic beef consumption increased [40,41]. What consumers perceive and what leads them to behave differently during animal diseases are worth exploring.

With its advantages of being low in fat and high in protein, chicken products have become more and more important, and their production and consumption are growing increasingly. According to the Food and Agriculture Organization (FAO), poultry output was 94.2 million tons in 2013 around the world. As estimated, poultry production will rise by 120% from 2010 to 2050, and it would replace pork as the most globally consumed type of meat [42]. China accounts for 15.6% of the global output of poultry [43]. Therefore, the quality and safety of chicken products in China affects not only the health and safety of Chinese consumers, but also the chicken products market worldwide. Data from FAO reveal that, from1985 to 2012, China’s annual consumption growth rate for chicken has surged from just 7.46% to 19%, second only to pork. In 2014, Chinese chicken production amounted to 13080 thousand tons, accounting for 15.15% of the world’s chicken production. Provinces located in the southern region of China, such as Guangdong or Jiangsu, have a great number of chicken products in production, consumption and trade according to the 2011 China Rural Statistical Yearbooks. In 2011, top five cities of the poultry production are: Shandong (254.5 tons), Guangdong (150.3 tons), Jiangsu (138.9 tons), Guangxi (128.8 tons), Liaoning (125.2 tons). Currently, for most families, eggs are essential food in daily meals, especially breakfast. In Guangdong, there is a saying—“No chicken, no feast”—indicating that consuming chicken products has become a common habit. Nevertheless, avian influenza has brought great destruction to the poultry industry and heightened public awareness of food safety. In May 22nd, 2013, Juan Lubroth, the Chief Veterinary Officer of FAO, said that the economic impact of H7N9 avian influenza is stunning, causing the agriculture sector to lose more than $65 billion dollars. China has suffered a great loss as well. According to the 2004–2013 Chinese Rural Statistical Yearbooks, in 2013, China’s poultry production growth rate was negative for the first time in ten years, according to the 2004-2013 China Rural Statistical Yearbooks. In 2004 the national poultry production was 13,510,000 tons, in 2010 the number was up to 16,561,000 tons. And in 2011 to 17,088,000 tons, in 2012 to 18,230,000 tons, in 2013 it was just 17,980,000 tons. Meanwhile, data provided by the China Animal Husbandry Association show that, due to H7N9, China’s poultry industry annual economic losses were up to 1000 billion Yuan during 2013. The destruction has affected more than 40 million poultry farmers, thousands of enterprises and nearly 1 million people in the industry chain.

While most of the previous research has emphasized the avian influenza virus from pathology perspective, and the past decades have witnessed a few studies concentrating on evaluating thoroughly the various economic impacts of animal diseases in other countries [44,45,46], little attention has been paid to examining impacts from the perspective of social economic and management of animal diseases for consumers in China. This paper attempts to focus on purchase intentions toward chicken products during avian influenza in China, in order to get a better picture of the complex factors involved.

This paper seeks to assess factors affecting consumers’ purchase intentions toward poultry products regarding avian influenza. Based on a representative sample of 330 consumers in Guangzhou, China, our study is designed to explore the influencing factors of their distrust, and outline solutions to eliminate these concerns regarding avian influenza. We focus specifically on Guangzhou, mainly for the following 3 reasons. Firstly, as mentioned above, cities located in southern regions have a large amount of chicken production, consumption and trade, according to the 2011 China Rural Statistical Yearbooks, in 2011, top five cities of the poultry production are: Shandong (254.5 tons), Guangdong (150.3 tons), Jiangsu (138.9 tons), Guangxi (128.8 tons), Liaoning (125.2 tons). As the capital city of Guangdong, Guangzhou should not be overlooked. Secondly, eating live chicken is a habit that formed over a long time, especially for older people in Guangzhou, implying that the impact of the invasion of avian influenza for Guangzhou consumers is evident. Thirdly, scientists found that the virus gene isolated from the H7N9 human case was similar to that of the virus isolated from chicken flocks in the live poultry market and concluded that it was a direct source of human infection [47]. Poultry production and sales have led to the spread of H5N1 and other avian influenza viruses [48]. After closing the live poultry market, the spread of avian influenza has been quickly controlled [49,50]. How government policies will work in both preventing avian influenza and satisfying consumers is worth exploring.

## 2. Data and Methods

### 2.1. Survey Questionnaire

In 2017, a total of 420 questionnaires were distributed to potential chicken purchasers at food markets and supermarkets in Guangzhou, by the face-to face interview method. As a result, 371 questionnaires were returned, of which 330 were correctly filled out (78.3% response rate). Data were collected on gender, age, educational level, and household information such as number of family members and income. In addition, 26 questions were asked about the variables of household consumption and consumer motivations towards purchase intensions, the subjective norm, the information perception, the risk perception and the general consumer confidence including optimism and pessimism. Measurement items were all adopted and developed from established studies [4,5,6,7,8,9,10,11,12,13,14,15,16,17,18,19,20,21,22,23].

Data were collected on household consumption and consumer motivations. Consumers were asked how much they would be willing to pay for the iced fresh chicken at that market. Specific questions were asked about the influence of the policies of the government, media and their friends toward iced fresh chicken hazards and thus their purchase intention and the influence on their own attitude. Lastly, consumers were asked about the convenience, brand, package design and safety certificate of these iced fresh chickens.

Of all valid survey participants, females comprised 62.12% and 29.10% of the 330 respondents were in the range of 18 to 25 years old, 38.18% aged from 26 to 40, 20.00% aged from 41 to 55, and 12.73% above 56 years old. In terms of education level, those educated to high school (or secondary) level were the highest proportion—31.51%—while junior high school was the second largest, accounting for 30.30%. When it comes to household income, the average monthly household income is medium, and the rate below 7500 Yuan a month accounts for 80.30%. Finally, the number of family members being 5 or more accounts for 43.03% of the overall sample, followed by 4, accounting for 28.79%.

### 2.2. Method and Variable Selection

The ordered Probit/Logit model regarding purchase intentions as ordinal variables was selected as the analytic method. The items were rated on 5-point Likert scales, ranging from ‘disagree strongly’ (1) to ‘agree strongly’ (5). If using the OLS regression method, the data will be handled as cardinal numbers, assuming that purchase intentions follow a Normal Distribution. Therefore, the selection of an ordered Probit/logit model is better.

We not only follow the public risk perception model [11] and the model of consumer confidence in food safety [21], but also consider the actual situation of consumers. Finally, this paper focuses on the following 7 attributes affecting consumers’ purchase intentions: (a) Individual basic characteristics, (b) concerns about the outbreak of avian influenza, (c) attitude toward purchase intentions, (d) subjective norm, (e) information perception, (f) risk perception, (g) general consumer confidence including optimism and pessimism (See Table 1). The variables are explained in detail as follows:

(a) The dimension of individual basic characteristics, including gender, age, education level, household income and family size. Gender is a dummy variable, namely female = 0, male = 1.

(b) The concerns about the outbreak of avian influenza. This dimension includes frequency of consuming chicken products in a week, the ratio of chicken product consumption to total meat consumption in the family and the degree of concern about avian influenza.

(c) Attitude toward purchase intentions. It has been identified as how an individual feels about performing a behavior, which is determined through beliefs about the consequences when one is performing a behavior and an evaluation, either positive or negative, of the desirability of these consequences [5,6].

(d) Subjective norm. Subjective norm has also been discussed in previous studies. Subjective norm can be defined as the perception of an individual about how much people one cares about agree or disagree his/her behavior [5,6,51].

(e) Information perception. A major barrier to food safety procedures in developing countries may be the level of available knowledge relating to food quality and safety [52,53]. As technology develops, information may be disorienting, such as contamination and additives of food [54,55].

(f) Risk perception. In past studies, risk perception has also been discussed as an important factor in consumer confidence in food. Perceived risks are related to both morbidity and mortality, along with factors such as unfamiliar, uncertain, unknown, uncontrollable, and severe consequences associated with risk perception [56].

(g) General consumer confidence is identified as to what extent consumers will perceive that food is generally safe and does not cause any harm to their health or to the environment. It consists of optimism and pessimism. Optimism indicates that consumers are satisfied about the safety of food and think that food is generally safe. Pessimism indicates that consumers worry and are suspicious about the safety of food [22,57].

Due to consumers’ purchase intentions toward poultry products based on an ordinal ranking in five categories, an ordered logit model is used. For the i-th individual, the model can be written as:

(1) $$Y_{i}{}^{*}={X_{i}}^{\prime }\beta +\varepsilon_{i}\;\mathrm{if}\;r_{j-1}<Y_{i}{}^{*}\leq r_{j}\;\mathrm{Where}\;j=1\;\mathrm{to}\;4,\;i=1,\;2,\ldots ,330$$

(2) $$\mathrm{Ultimately},\;\mathrm{the}\;\mathrm{establishment}\;\mathrm{of}\;\mathrm{ordinal}\;\mathrm{Logit}\;\mathrm{model}\;\mathrm{is}\;\mathrm{expressed}\;\mathrm{as}\;P(y=j|x)=\frac{1}{1+e^{-(\alpha +\beta X_{i})}},i=1,2,\ldots 8$$

According to the above model, the observed variable $Y_{i}$ arises from $Y_{i}{}^{*}$ and it assumes five levels separated by four cut-points as specified below:

(3) $$Y_{i}=1\;\mathrm{if}\;Y_{i}{}^{*}\leq r_{1}$$

(4) $$Y_{i}=2\;\mathrm{if}\;r_{1}<Y_{i}{}^{*}\leq r_{2}$$

(5) $$Y_{i}=3\;\mathrm{if}\;r_{2}<Y_{i}{}^{*}\leq r_{3}$$

(6) $$Y_{i}=4\;\mathrm{if}\;r_{3}<Y_{i}{}^{*}\leq r_{4}$$

(7) $$Y_{i}=5\;\mathrm{if}\;r_{4}<Y_{i}{}^{*}$$

where Y is purchase intentions toward poultry products, $X_{1}$ represents the basic characteristics of individual dimensions, $X_{2}$ is the degree of concern on the outbreak of avian influenza, $X_{3}$ acts as the attitude,$\;X_{4}$ the subjective norm dimension, $X_{5}$ the information perception $X_{6}$ risk perception, $X_{7}$ the optimism, $X_{8}$ the pessimism, $\beta =(\beta_{0},\beta_{1},\beta_{2},\beta_{3},\beta_{4},\beta_{5},\beta_{6},\beta_{7},\beta_{8})$ are variable coefficients to be estimated, and εis for the random error. 17 measurement variables (See Table 1) were selected for explanatory variables. Table 2. describes the average scores and standard error of the measured questionnaire items.

## 3. Results and Discussion

On the basis of the results of the regression, Table 3 displays the marginal effects of consumers’ purchase intentions for chicken products, reflecting changes in the independent variable due to the variable effects of different results of probability. Overall, Pseudo R^2^ of the equations is 0.24, respectively, indicating that the model has strong explanatory power. In Table 3, the four categories of cut-points are also reported. As a rule, cut-points are treated as nuisance parameters necessary for estimation purposes, but without an intrinsic meaning [58].

From the consumers’ attitude perspective, a positive and statistically significant effect is found, indicating that the more consumers believe purchasing chicken products may pose a threat to their health, the more they will reduce purchasing chicken products. Based on the estimated marginal effect, the probability that consumers agree to a reduction in purchasing chicken products increases by 6.2% if they believe purchasing chicken products may have threatened their health during the period of avian influenza. In addition, consumers believe that during the period of avian influenza, they will, to some extent, reduce their purchasing of chicken products. The marginal effect estimates that the probability of consumers reducing eating chicken products to some extent increases by 1%, consumers agree to a certain extent that reducing the purchase of chicken products will decrease by 2.3%, and those who agreed will increase 16.2%. This result may be explained as health consciousness [59], which indicates to what extent concern is integrated into the daily activities of an individual. Because consumers consider health as an important parameter while purchasing food, and reveal interest in issues associated with food and health [60,61], they will intentionally avoid the purchasing of chicken to prevent the risk of food disease. A consumer’s choice is influenced by many factors in which health concern has been given more weight than other factors like concern about the environment and food/diet [32]. Total probability of the five categories of the dependent variable is 1.0. The marginal impacts add up to 0.0, shown in Table 3, because a change in one category probability must be compensated for exactly by changes in the probabilities of other categories.

In terms of the subjective norm, the influence of media reports on consumers is positively associated with purchase intentions and significant at the 10% level of significant effect. Marginal effect estimates showed that the probability that media reports will influence whether consumers should buy chicken products increases every 1%, consumers agree that a price increase in chicken will see a 6.1% decrease in sales. This result is in accordance with previous studies [62,63], indicating that because they are out of reach when assessing food safety before consumption, consumers must rely on information provided by others, such as the media. However, as time passes, mass media is playing an important role in both building and undermining consumer confidence. De Jonge, Van Trijp, Renes and Frewer (2010) addressed that daily media reports, such as newspapers, receive a lot of attention and affect general consumer confidence significantly [22]. They figured out that, during 2003-2006, avian influenza dominated newspaper coverage, which is proven to be positively associated with consumer recall of food safety incidents. The result is consistent with previous studies in that information through mass media associated with food contamination or outbreaks of animal diseases may cause consumers to change their preferences for meat consumption [37,64,65].

For consumer attributes relating to information perception, the origin of the chicken products is essential to consumers. Marginal effects estimates indicate that the probability of a consumer to agree to a certain extent to reduce the purchase of chicken product increases by 7.4% if consumers view the origin of chicken products as important. The possible explanation for this may be that avian influenza is known to be transmitted from wild birds to chicken, suggesting that the safety and cleanliness of the origin of chicken products is vital to consumers. In this literature, H1N1 was called swine flu at the beginning, which is a confusing message and misled consumers and caused great uncertainty [66]. Nevertheless, Van Wezemael et al. [34] bserved a conflicting phenomenon that some consumers felt there is a lack of information about beef safety available, while others seemed to be faced with an overload of information, which might also increase the difficulties in assessing beef safety [34]. As a result, the government should arm the public with information which would allow people to make informed decisions, reducing their exposure to contaminants [27].

From the risk perception perspective, a positive and statistically significant effect is found that, the more consumers believe purchasing chicken products is a risk to a certain extent in the period of avian influenza, the more they will reduce their purchase of chicken products. The estimated marginal effect estimates that the probability that consumers agree to reduce purchasing chicken products increases 8.6% if they believe purchasing chicken products is a risk to a certain extent during the period of avian influenza. Consistent with previous studies, perceived risks are an important construct underpinning purchase intention [12,13].

Pessimism is tested as an influencing factor. A positive and statistically significant effect is found for ‘Worry about the safety of chicken products in the period of avian influenza’. The marginal effect estimation results show that, during the period of avian influenza, the probability of consumers somewhat agreeing to reduce the purchase of chicken products increases 7%, if they feel uneasy. The sign of ‘Suspicious about the safety of chicken products in the period of avian influenza’ is positive and significant at the 10% level, with its marginal effect estimation results revealing that, during the period of avian influenza, the probability of consumers being skeptical increases by 1% and the probability of consumers agreeing to reduce their purchase of chicken products will increase by 14.9%. These results indicate that, during the period of avian influenza, consumers were less confident, but were concerned and skeptical about chicken products’ quality and safety.

## 4. Conclusions

China’s increasing food safety problem is attracting national attention and awareness [28,67,68], and concerns over consumers’ health and the quality and nutritional value of food are emerging [69,70]. Based on survey data from 330 consumers in Guangzhou, the overall goal of this study was to analyze the factors that affect purchase intentions toward chicken products during avian influenza. According to previous studies, 7 factors such as individual characteristics, concern level, attitude, subjective norm, information perception, risk perception and general consumer confidence—which includes optimism and pessimism—are expected to affect the purchase intentions of consumers in Guangzhou. We analyzed the effect of these factors by using an ordinal Logit model. We found that possible health threat, media reports, the origin of chicken products, perception of the potential risk of being infected by avian influenza and pessimism have a statistically significant relationship with purchase intentions. Among them, consumers’ expectations for the quality and safety of chicken products significantly affect consumers’ willingness to purchase chicken products. During the avian flu outbreak period, the more consumers feel that chicken products pose a threat to their health, the lower their intention to buy chicken products. That is to say, in the context of avian flu, consumers generally have low levels of trust in the quality of chicken meat. They believe that buying fresh chicken may pose a health hazard. Therefore, during the avian flu outbreak, due to the high risk of fresh chicken products and the high threat to health, the uncertainty in the purchase transaction is increased, and consumers will reduce the purchase of chicken products to a certain extent. Second, the mass media will influence and change consumers’ willingness to purchase chicken products through the dissemination of information about, for example, avian flu and animal diseases and food contamination. In the context of avian flu, relevant information is transmitted to the public by the media, affecting and changing consumers’ preferences for meat consumption. At the same time, consumer’s information perception, risk perception and pessimism will affect consumers’ willingness to buy under avian flu. The effects of the other factors on purchase intentions were not confirmed in our study.

The conclusions of this study provide a reference on consumer preferences for the governments. First, the government should take the lead and coordinate with all parties to establish a relatively complete public health system to ensure the stable and healthy development of the poultry meat products market, from the corresponding infrastructure, institutional arrangements, staff, and the public in all aspects of the poultry industry. The promotion of publicity and the introduction of relevant laws and regulations, and so forth.

In detail, in terms of infrastructure, for large farms they should be registered for the record and regular inspected; for decentralized feeding households, a cooperative function should be run. More specific, relevant staff is needed to perfect the registration of a regular inspection and to provide free or preferential treatment of chicken breeding site disinfection, drug treatment, vaccination programs [71]. Besides, laws with punitive measures for selling clinically sick chickens are critical [72]. Rational and scientific governance of the industry will restore consumers’ willingness to purchase and consumer confidence in the context of avian flu. At the same time, the government should develop the chilled chicken industry according to local conditions and guide consumers to purchase with changes in the consumption habits of live chickens. Changes will help reduce the risk of avian flu transmission and increase consumers’ willingness to purchase chicken products in the context of avian flu.

## 5. Implications

Moreover, we suggest that in order to reduce the general public’s scares and worries, it is important to provide precise information through public media should not either be insufficient or overloaded [34]. At present, the dissemination of online public opinion on food safety has resulted in very serious problems, which may have adverse impacts [11]. Therefore, it is also important to promote basic knowledge of chicken products safety and quality among consumers and enhance the public knowledge about avian influenza in China.

Finally, our results show that experts and the media should disseminate scientific information about chicken products during avian influenza and curb misleading reports. The power of social media amplification is critical. For example, consumer risk perceptions increased and consumer benefit perceptions diminished with high reporting levels about genetically modified food [73]. When a consumer is continually exposed to the same message, it will be easier to retrieve the message compared to those messages has not been exposed often. What’s more, there is no conclusive evidence that H7N9 avian influenza virus can be transmitted from the birds directly to humans so far. Hence, experts and the media should shoulder their social responsibility, providing more useful information, but not to make up facts without foundation to attract the attention of the public’s eyeballs during infectious diseases.

## Figures and Tables

**Table 1:** Variables.

Dimension	Code	Measurable Variable Dimension
Concerning	CON	The degree of concern about avian influenza
	FRE	Frequency of eating chicken products a week of the family
	PRO	The proportion of chicken products consumption accounted for total consumption of meat products in the whole family
Attitude	ATT1	Purchasing chicken products during the outbreak of avian influenza makes me feel uncomfortable due to the possibility of harming our health
	ATT2	Will reduce purchasing chicken products during the outbreak of avian influenza
Subject Norm	SN1	Influence on purchasing chicken products from family or friends
	SN2	Influence on purchasing chicken products from media reports about the public’s behavior
	SN3	Influence on purchasing chicken products from government policies
Information Perception	IP1	Influence from information on chicken products regarding their origins
	IP2	Influence from information on chicken products regarding their nutritional situation
	IP3	Influence from information on chicken products regarding their quality
Risk Perception	RP1	Consumption of chicken products will increase the chance of you getting sick in the period of avian influenza
	RP2	Purchasing chicken products is a risk to a certain extent in the period of avian influenza
Optimism	OP1	Be optimistic about the quality and safety of chicken products in the period of avian influenza
	OP2	In general, chicken products are safe in the period of avian influenza
Pessimism	PE1	Worry about the safety of chicken products in the period of avian influenza
	PE2	Suspicious about the safety of chicken products in the period of avian influenza
Purchase intention	PI	The degree of purchase intentions toward poultry products

Note: CON refers to Concern. FRE refers to Frequency. PRO refers to Proportion. ATT1 refers to Attitude1 while ATT2 refers to Attitude2. SN1, SN2 and SN3 refer to Subject Norm1, Subject Norm2 and Subject Norm3 respectively. IP1, IP2 and IP3 refer to Information Perception1, Information Perception2 and Information Perception3 respectively. RP1 and RP2 refer to Risk Perception1 and Risk Perception2. OP1 and OP2 refer to Optimism1 and Optimism2. PE1 and PE2 refer to Pessimism1 and Pessimism2. PI refers to Purchase Intention.

**Table 2:** Item average scores and standard error values.

Variables	Options	Average	Standard Error
CON	1 = disagree strongly, 2 = disagree, 3 = neither, 4 = agree, 5 = agree strongly	4.06	0.88
FRE	1 = disagree strongly, 2 = disagree, 3 = neither, 4 = agree, 5 = agree strongly	3.00	1.66
PRO	1 = disagree strongly, 2 = disagree, 3 = neither, 4 = agree, 5 = agree strongly	2.11	0.97
ATT1	1 = disagree strongly, 2 = disagree, 3 = neither, 4 = agree, 5 = agree strongly	3.68	1.38
ATT2	1 = disagree strongly, 2 = disagree, 3 = neither, 4 = agree, 5 = agree strongly	4.07	1.24
SN1	1 = disagree strongly, 2 = disagree, 3 = neither, 4 = agree, 5 = agree strongly	3.17	1.35
SN2	1 = disagree strongly, 2 = disagree, 3 = neither, 4 = agree, 5 = agree strongly	3.71	1.25
SN3	1 = disagree strongly, 2 = disagree, 3 = neither, 4 = agree, 5 = agree strongly	3.49	1.38
IP1	1 = disagree strongly, 2 = disagree, 3 = neither, 4 = agree, 5 = agree strongly	3.17	1.35
IP2	1 = disagree strongly, 2 = disagree, 3 = neither, 4 = agree, 5 = agree strongly	3.85	1.23
IP3	1 = disagree strongly, 2 = disagree, 3 = neither, 4 = agree, 5 = agree strongly	3.87	1.25
RP1	1 = disagree strongly, 2 = disagree, 3 = neither, 4 = agree, 5 = agree strongly	3.83	1.37
RP2	1 = disagree strongly, 2 = disagree, 3 = neither, 4 = agree, 5 = agree strongly	3.68	1.24
OP1	1 = disagree strongly, 2 = disagree, 3 = neither, 4 = agree, 5 = agree strongly	2.84	1.29
OP2	1 = disagree strongly, 2 = disagree, 3 = neither, 4 = agree, 5 = agree strongly	2.81	1.34
PE1	1 = disagree strongly, 2 = disagree, 3 = neither, 4 = agree, 5 = agree strongly	3.59	1.23
PE2	1 = disagree strongly, 2 = disagree, 3 = neither, 4 = agree, 5 = agree strongly	3.60	1.25
PI	1 = disagree strongly, 2 = disagree, 3 = neither, 4 = agree, 5 = agree strongly	3.93	1.19

**Table 3:** Estimated results and marginal effects of variables.

Code	Estimated Results	Marginal Effects				
		1	2	3	4	5
Personal						
GEN	0.130 (0.243)	−0.002 (0.003)	−0.003 (0.006)	−0.021 (0.040)	−0.005 (0.010)	0.031 (0.058)
EDU	−0.901 (0.137)	0.001 (0.002)	0.002 (0.004)	0.015 (0.023)	0.003 (0.005)	−0.021 (0.032)
AGE	0.152 (0.135)	−0.002 (0.002)	−0.004 (0.004)	−0.025 (0.022)	−0.005 (0.005)	0.036 (0.032)
INC	0.123 (0.110)	−0.001 (0.001)	−0.003 (0.003)	−0.020 (0.018)	−0.004 (0.004)	0.030 (0.026)
FAM	0.000 (0.117)	−0.000 (0.001)	−0.000 (0.003)	−0.000 (0.019)	−0.000 (0.004)	0.000 (0.028)
Concerning						
CON	−0.135 (0.117)	0.002 (0.002)	0.004 (0.004)	0.022 (0.023)	0.004 (0.005)	−0.032 (0.033)
FRE	0.061 (0.090)	−0.001 (0.001)	−0.002 (0.002)	−0.010 (0.015)	−0.002 (0.003)	0.014 (0.021)
PER	−0.020 (0.153)	0.000 (0.002)	0.001 (0.004)	0.003 (0.025)	0.001 (0.005)	−0.005 (0.036)
Attitude						
ATT1	0.260 * (0.143)	−0.003 (0.002)	−0.007 * (0.004)	−0.043 * (0.024)	−0.009 (0.007)	0.062 * (0.034)
ATT2	0.683 *** (0.150)	−0.008 ** (0.003)	−0.018 ** (0.006)	−0.113 *** (0.021)	−0.023 * (0.013)	0.162 *** (0.036)
Subject Norm						
SN1	−0.188 (0.124)	0.002 (0.002)	0.005 (0.003)	0.031 (0.021)	0.006 (0.005)	−0.044 (0.029)
SN2	0.257 * (0.150)	−0.003 (0.002)	−0.007 (0.004)	−0.042 * (0.025)	−0.009 (0.007)	0.061 * (0.036)
SN3	−0.152 (0.133)	0.002 (0.002)	0.004 (0.004)	0.025 (0.022)	0.005 (0.005)	−0.036 (0.031)
Information Perception						
IP1	0.314 ** (0.143)	−0.004 * (0.002)	−0.001 * (0.004)	−0.052 * (0.024)	−0.010 (0.007)	0.074 ** (0.034)
IP2	−0.640 (0.166)	0.001 (0.002)	0.002 (0.004)	0.011 (0.027)	0.002 (0.006)	−0.015 (0.039)
IP3	−0.031 (0.152)	0.000 (0.002)	0.001 (0.004)	0.005 (0.026)	0.001 (0.005)	−0.007 (0.036)
Risk Perception						
RP1	0.059 (0.114)	−0.001 (0.002)	−0.002 (0.003)	−0.009 (0.018)	−0.002 (0.004)	0.014 (0.027)
RP2	0.360 * (0.141)	−0.005 * (0.003)	−0.010 ** (0.005)	−0.058 ** (0.023)	−0.012 (0.008)	0.086 ** (0.034)
Optimism						
OP1	−0.124 (0.129)	0.001 (0.002)	0.003 (0.003)	0.020 (0.021)	0.004 (0.005)	−0.029 (0.031)
OP2	0.019 (0.140)	−0.000 (0.002)	−0.000 (0.004)	−0.003 (0.023)	−0.001 (0.005)	0.004 (0.033)
Pessimism						
PE1	0.030 * (0.171)	−0.004 (0.002)	−0.008 (0.005)	−0.049 * (0.024)	−0.010 (0.008)	0.070 * (0.040)
PE2	−0.631 *** (0.186)	−0.008 (0.003)	−0.017 ** (0.006)	−0.104 *** (0.032)	−0.021 (0.013)	0.149 *** (0.044)
Cut-point 1	1.111					
Cut-point 2	2.213					
Cut-point 3	4.290					
Cut-point 4	5.598					
$\;Prob>X^{2}$	0.0000					
$Pseudo\;R^{2}$	0.2386					

Note: GEN refers to Gender. EDU refers to Education. AGE refers to Age. INC refers to Income while FAM refers to Family. * Significant at 10%. ** Significant at 5%. *** Significant at 1%.
